# Evaluating the Effectiveness of Complexity Features of Eye Movement on Computer Activities Detection

**DOI:** 10.3390/healthcare10061016

**Published:** 2022-05-31

**Authors:** Twin Yoshua R. Destyanto, Ray F. Lin

**Affiliations:** 1 Department of Industrial Engineering and Management, Yuan Ze University, Taoyuan 32003, Taiwan; juifeng@saturn.yzu.edu.tw; 2 Department of Industrial Engineering, Universitas Atma Jaya Yogyakarta, Yogyakarta 55281, Indonesia

**Keywords:** human activity recognition, eye-movement features, complexity, multi-scale entropy

## Abstract

Recently, tools developed for detecting human activities have been quite prominent in contributing to health issue prevention and long-term healthcare. For this occasion, the current study aimed to evaluate the performance of eye-movement complexity features (from multi-scale entropy analysis) compared to eye-movement conventional features (from basic statistical measurements) on detecting daily computer activities, comprising reading an English scientific paper, watching an English movie-trailer video, and typing English sentences. A total of 150 students participated in these computer activities. The participants’ eye movements were captured using a desktop eye-tracker (GP3 HD Gazepoint™ Canada) while performing the experimental tasks. The collected eye-movement data were then processed to obtain 56 conventional and 550 complexity features of eye movement. A statistic test, analysis of variance (ANOVA), was performed to screen these features, which resulted in 45 conventional and 379 complexity features. These eye-movement features with four combinations were used to build 12 AI models using Support Vector Machine, Decision Tree, and Random Forest (RF). The comparisons of the models showed the superiority of complexity features (85.34% of accuracy) compared to conventional features (66.98% of accuracy). Furthermore, screening eye-movement features using ANOVA enhances 2.29% of recognition accuracy. This study proves the superiority of eye-movement complexity features.

## 1. Introduction

### 1.1. Significance of Computer Activity Recognition on Healthcare Issues

Nowadays, developing a tool or system that can detect and recognize human activities is quite prominent in providing some health issue prevention and even long-term healthcare for human beings [1,2]. The biological data comprising human heart rate [3], muscle activities [2], motion acceleration [4], and eye movement have been involved in developing certain assistive healthcare systems and technologies. The usage of these biometric data can help human beings to understand their real-time status during their daily activities.

In the current era, the typical human daily activities are various kinds of computer activities. The development of information technologies enables people to work more productively using computers. However, this kind of activity often causes healthcare issues, comprising musculoskeletal disorders that are caused by typing activity or improper working posture [5,6], and any vision syndrome (e.g., eye strain, dry eyes) because of long-term computer usage [7]. Therefore, the posture and working behavior need to be adjusted according to the task that is being worked on. To understand the best working posture and behavior, the system first needs to have the capability of recognizing the user’s computer activity. To fulfill this need, once again, biological data can be utilized in developing the activity detection model.

### 1.2. Eye-Movement Complexity Features for Activity Detection Models

Previous studies acknowledge that eye-movement data has been used to build certain activity detection models involving Artificial Intelligence (AI) methods [8,9,10]. The eye movement data captured by the eye-tracker, comprising eye-fixation, blink, pupil diameters, and saccade, are used to generate certain eye-movement features. Conventionally, eye movement metrics are static since it just calculates a single specific aspect of vision. Therefore, it ignores the multiple time scales inherent in such time series [11]. These conventional eye-movement features have been used for building machine learning or deep learning models to detect computer activities. For example, Bulling et al. [9] generated 19 conventional eye-movement features from eyes saccade, fixation, and blink, tracked using electrooculography (EOG), to build computer activity detection models using Support Vector Machine (SVM). Their models resulted in 72.7% of accuracy in detecting reading, browsing, writing, watching a video, and copying words. Our previous study [8] also utilized 19 eye-movement features to build AI models to detect reading, watching videos, and typing activities. The models were developed using Convolutional Neural Network (CNN), and the 19 eye-movement features were calculated based on raw eye fixation, pupil diameter, blink, and saccade, tracked using desktop-eye-tracker. The models had an accuracy between 42.78 and 93.15% (mean: 76.14%) in detecting reading, watching videos, and typing activities.

Specific eye-movement features were reported to help the built model recognize the activities [8]. For example, watching videos and typing activities are best detected using pupil and blink-related features [8,12,13]. To detect the reading activity, pupil dilation [13,14] related features are helpful because pupil activities have a high correlation with cognition and perception [15]. However, these conventional eye-movement features are limited and sometimes fail to describe the pattern of different activities consistently. The conflict appeared between the results of our previous study [8] and the results from Bulling et al. [9]. The eye-movement features related to fixation were useful to detect the typing activity in Bulling et al. [9] but not in our previous study [8]. Therefore, further analysis of the conventional eye-movement features is needed to have clearer eye-movement data patterns for distinguishing different computer activities.

Meanwhile, complexity analysis [11] was recently used in certain human biometric data comprising heart rate [16], cerebral hemodynamics [17], blood pressure [18], and infants’ limb movements [19]. The use of the complexity of these biological data can describe the human states related to their health conditions [16,17,18] and activities [19,20]. The benefit is the potential application to eye-movement features. The result may help AI models built by using eye-movement complexity features to distinguish different human activities [17] which then may raise the models’ accuracy in detecting the activities. However, based on our experience, none of the complexity analysis-related research used eye-movement features to do any human activity recognition.

### 1.3. Research Objectives

The conventional eye-movement features mentioned have certain limitations in helping the AI models detect computer activities. On the other hand, the complexity analysis may have potency in describing the changes in the eye-movement pattern during different computer activities. Therefore, this study aimed to evaluate the performance of eye-movement complexity features compared to conventional eye-movement features, in detecting the computer activities, comprising reading an English scientific paper abstract, watching an English movie-trailer video, and typing English sentences. Both complexity and conventional eye-movement features were evaluated using analysis of variance (ANOVA) of the General Linear Model that treated participants as random factors and computer activities as fixed factors, to build three kinds of machine learning (ML) models comprising Support Vector Machine (SVM), Decision Tree (DT), and Random Forest (RF).

## 2. Materials and Methods

### 2.1. Participant, Apparatus, and Materials

One hundred and fifty colleges and graduate international students from Yuan Ze University (all of them are Southeast Asian) were recruited (by using a local announcement) and participated in this study, voluntarily. They were between 20 and 27 years old (mean = 23.52) with the same proportion of female and male composition. Throughout the experiment, all participants reported having normal or corrected-to-normal vision, and no color-related vision abnormality was reported.

**Figure 1:**
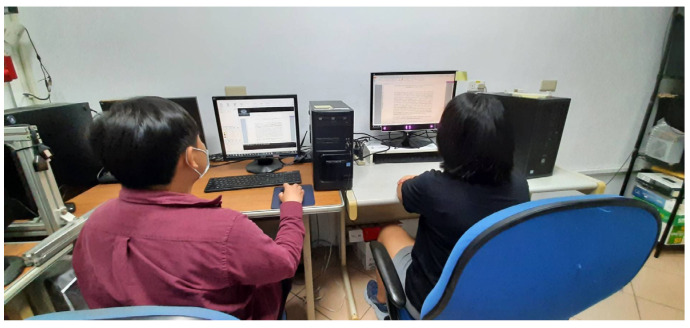
Apparatus and participant setting during the experiment.

The apparatus used in this study included a GP3 HD (Gazepoint™ Canada) 150Hz desktop eye-tracker, an Intel^®^ Core™ i7-6700 built-in personal computer (PC), two screen monitors with 1280 × 1024 display resolution, and a 720p resolution of a webcam (Logitech^®^ C270). The Gazepoint Analysis™ software installed on the PC was used to operate the eye-tracker and record the collected data. The activities during the experiment were recorded using a webcam. Each stimulus was shown on a screen in front of the participant, and the other screen was used to operate the computer and run the installed related software. These apparatuses were set in a controlled condition room, with approximately 600 lx of illuminance and 23–24 °C temperature, as shown in Figure 1.

Experimental stimuli were a video, English sentences, and an English journal paper. The video, a movie trailer (https://bit.ly/watch-stimulus accessed on 5 February 2020) with approximately 64 dB, was used for the watching; the English sentences, taken from Liu et al. [21], were used for the typing activity; and the journal paper [22] was used for the reading activity.

### 2.2. Experimental Setting and Task

**Figure 2:**
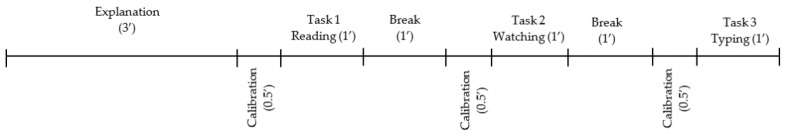
Experiment process from explanation to the third task.

During the experiment, as shown in Figure 1, the participant sat on the right side at a distance of approximately 55–65 cm from the screen monitor. In front of the monitor, the eye-tracker was placed to record the participant’s eye movements. The experimenter, who sat on the left side, explained verbally and in writing the experiment objective and procedure to the participant before beginning the experiment. The experimenter showed the stimuli to the participant and confirmed whether the participant was able to watch or read the stimuli. During the explanation, the participant was allowed to speak to ask questions, but the experimental tasks were performed without talking. However, as described in the explanation session, the participant was able to stop the experiment at any time. Before participating in the experiment, all participants signed a consent form allowing their data to be used for research purposes. As shown in Figure 2, the explanation took about three minutes and was followed by a half-minute eye-tracker calibration. The participant was asked to keep his/her head in a certain position to ensure the eyes were well captured by the eye tracker. However, the participant’s head was not restricted. This procedure was applied to get accurate and precise eye-movement data from daily activity [23]. After the calibration, the participant performed a one-minute experimental task and took a one-minute break. The procedure was repeated three times as the participant subsequently performed reading, watching, and typing tasks. The breaks were arranged to reduce fatigue, whereas the learning effect was ignorable because all the participants were familiar with the three computer activities. Considering the participants were international students enrolled in English-taught courses, and having at least intermediate English proficiency, therefore they were deemed eligible.

### 2.3. Data Preprocessing and Feature Selections

The eye movement data collected by the eye-tracker were processed using Gazepoint Analysis™ software (Gazepoint, Vancouver, BC, Canada). The processed data consisted of fixation X- and Y-coordinate positions (FPOGX, FPOGY), fixation duration (FPOGD), left pupil diameter in both pixels (LPD) and millimeters (LPMM), right pupil diameter in both pixel (RPD) and millimeters (RPMM), blink duration (BKDUR), blink frequency (BKPMIN), saccade distance (SAC_MAG), and saccade direction (SAC_DIR). The processed data had 93.56% mean and standard deviation 0,04 of gaze-data validity; therefore, they were eligible to be used for further steps [23,24]. All these processed data were then calculated to generate seven statistical parameters, comprising min, max, median, mean, standard deviation, variance, and skewness [25,26,27], resulting in 56 features. A three-second interval was used to separate the processed data and calculate the statistical parameters. Therefore, the data collected from a one-minute experimental task resulted in 20 data sets for each statistical parameter (e.g., mean, max, min, etc.). These data then are called the “conventional eye-movement features.” The all-conventional eye-movement features were then screened using ANOVA, with Minitab 18 (Minitab Ltd., Coventry, United Kingdom) as the statistical package tool.

Besides the conventional eye-movement features, the processed data were also decomposed to get the number of intrinsic mode functions (IMFs) by applying the decomposition of empirical mode (EMD) calculation [28,29]. IMFs consisted of limited simple function series from raw data *X(t)* that were filtered in the EMD process. The phase of refining the raw data *X(t)* was processed by decomposing them into IMFs, summed up, and last the leftovers as the formula that is stated in Equation (1).

(1) $$\hat{X}\left(t\right)=\overset{n}{\underset{i=1}{\sum }}c_{i}\left(t\right)+r_{n}\left(t\right)$$

where n = the total of IMFs number; $c_{i}$ = the ith IMFs; $r_{n}$ = the nth residue [28]. EMD operates without a predefined cut-off frequency as a filter bank [29] and can be used as a filter series of noise [30].

Each generated IMF from each raw data was then processed to get the multi-scale entropy (MSE). MSE was initiated by Costa and Goldberger [31] to understand the complexity of time series data from certain biological signals of the human heart rate. MSE consists of two subsequence steps called coarse-graining operation and sample entropy calculation. To do the coarse-graining operation for time series y with scale factor τ under the condition 1 ≤ j ≤ N/τ, the used formula is shown in Equation (2) below [21].

(2) $$y_{J}^{\tau }=\frac{1}{\tau }\overset{j\tau }{\underset{i=\left(j-1\right)\tau +1}{\sum }}x_{i}$$

where N indicates the amount of dataset, while $x_{i}$ represents the data points in the original time series. Then, the coarse-grained results were processed to get the sample entropy by applying Equation (3) below [32].

(3) $$SampEn\left(N,m,r\right)=-ln\frac{A^{m}\left(r\right)}{B^{m}\left(r\right)}$$

where *m* denotes the consecutive data points number, *r* represents the tolerance of accepting the match, *B* indicates the number of vectors *X_m_(j)* within *r* of *X_m_(i)*, and *A* denotes the number of vectors *X*_m+1_*(j)* within *r* of *X*_m+1_*(i)*. Wolf et al. [33] mentioned that theoretically, possible and logical estimation for the probabilities can be achieved by setting the value of *N* at least $10^{m}$ to $30^{m}$ points [21,34,35]. Then to determine the value of *r*, it followed the recommendation of Costa et al. [36] with the *r* range as 0.1~0.2 times the standard deviation (*SD*) of original raw time series data. Therefore, to calculate the MSE in this study, *m*, *r*, τ were determined as 2, 0.15*SD*, and 10, respectively [21,36]. As in conventional eye-movement features, the all-generated complexity eye-movement features were then screened using ANOVA, to get the important features.

### 2.4. AI Modelling for Computer Activities Detection

To compare the effectiveness of ML methods, the statistical screening test, and the complexity method, three ML methods, comprising Support Vector Machine (SVM), Decision Tree (DT), and Random Forest (RF), were applied to four data sets to build 12 models. The four data sets were the combinations of screened and unscreened features and conventional and complexity features. 

The SVM models were built using default values of modeling parameters based on scikit-learn 1.0.2 [37,38]. The models from DT were developed using a default random state, with a maximum depth of 10 and minimum samples leaf 7. The RF models were built using 5 maximum depths and 1000 trees in the forest [39]. The summary of AI models’ architecture is shown in Table 1 below. A total of 80% of the dataset was used as training, and 20% was used as a testing part. The training and testing dataset were selected randomly. Each AI model had six replications to get the average and standard deviation of recognition accuracy.

## 3. Results

### 3.1. Important Eye-Movement Features Screened Using ANOVA

All 56 conventional eye-movement features were then tested using ANOVA. In the ANOVA tests, *p*-values of 0.05, 0.01, and 0.001 were set as the thresholds to screen the critical features. The screened features are shown in Table 2, in which a total of 45 features (items with ‘*’) were found critical—the computer activity had significant effects on these critical features.

Moreover, the MSE analysis resulted in 550 numbers of features, and then they were screened using ANOVA, which resulted in 379 numbers of features. These features were called the “eye-movement complexity features.” The summary of the screened features, the complexity index (CI), is shown in Table 3 below. CI is the sum of sample entropy value from time scale *i* = 1 to τ, that describes the system integrated complexity [40].

### 3.2. Computer Activities Detection Models Performances

**Figure 3:**
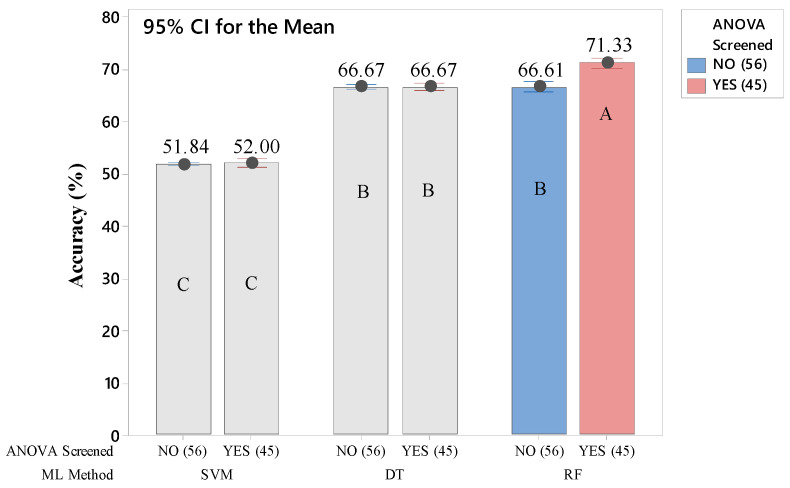
Accuracy comparison of 6 types of ML models built using conventional eye-movement features. The number of used features for building the models is indicated by numbers in parentheses. Means that do not share a letter are significantly different.

Each of the twelve ML models was replicated six times. For the conventional eye-movement features, six models were built and the results from important feature groups show that the average accuracy from six replications for SVM, DT, and RF were 52.00%, 66.67%, and 71.33%, respectively. As shown in Figure 3, the accuracy of the RF model built using ANOVA screened features was significantly higher (*p*-value < 0.001) compared to the accuracy of the RF model built using all features (66.61%). This significant difference in accuracy was shown using unshared letters inside the bars. The result of the SVM model built using screened features obtained the highest accuracy among the models built using conventional eye-movement features. On the other hand, the results from SVM and DT models built using important features were not significantly different compared to the accuracy from SVM and DT models built using all features (51.84% and 66.67%, respectively). This significant difference in accuracy was shown using shared letters inside the bars. The confusion matrices in Table 4 describe the different prediction results between ML models built using all conventional eye-movement features and important conventional eye-movement features only (represented by RF models, due to higher accuracy results). Values in tables are mean with *SD* in parentheses from the average of six RF models’ replications. The better accuracy should show higher values in diagonal cells with 100% maximum values and zero *SD*. The higher values show the bolder red highlight that indicates the model is able to predict the computer activity from testing data more accurately compared to the lighter highlighted one.

The comparison in Table 4 helps to understand how the important features play a significant role in distinguishing computer activities. The confusion matric of the RF model built using all conventional eye-movement features shows that the model confused to predict reading activity to watching activity (1.90%). After the features were screened, the accuracy to predict the reading activity significantly raised to 11.32%. Although the value is low, it was able to improve the model accuracy significantly, from 66.61% to 71.33%. The confusion matrices from DT and SVM models were not provided here because the results from these models were not significantly different.

**Figure 4:**
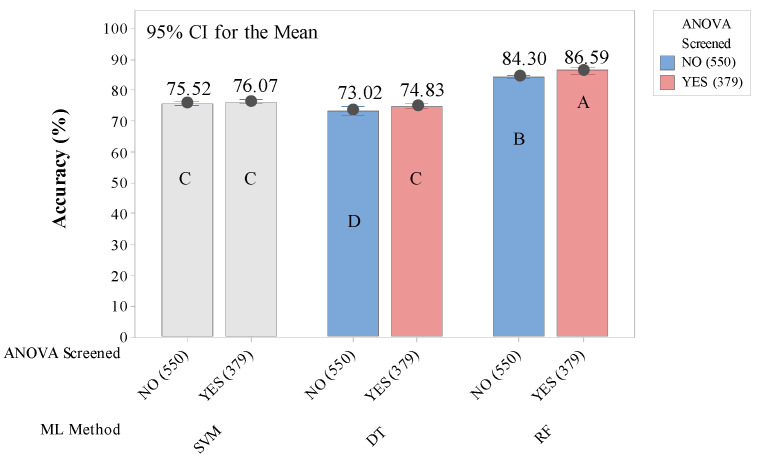
Accuracy comparison of 6 types of ML models built using eye-movement complexity features. The number of used features for building the models is indicated by numbers in parentheses. Means that do not share a letter are significantly different.

Meanwhile, in eye-movement complexity features groups, there were also six ML models each of which was run six times for replications. The prediction accuracies of the AI models comprising SVM, DT, and RF, built using all eye-movement complexity features were 75.52%, 73.02%, and 84.30% on average, respectively. As shown in Figure 4, a significant improvement in the average accuracies was obtained in DT (*p*-value < 0.05) and RF (*p*-value < 0.01) models with 74.83% and 86.59% accuracies, respectively. Similar to the results in the conventional eye-movement features group, the screened important features in the complexity group did not show significant improvement compared to the SVM model built using all eye-movement complexity features, with a 76.07% accuracy average. The confusion matrices for the models showed accuracy improvement (Table 5) and gave detailed descriptions that the important eye-movement complexity features helped the DT and RF models to increase the ability for predicting the reading activity (18.81 % to 19.97% in DT and 22.18% to 23.89% in RF). The confusion matrices from the SVM models were not provided here, because the results from these models were not significantly different.

**Figure 5:**
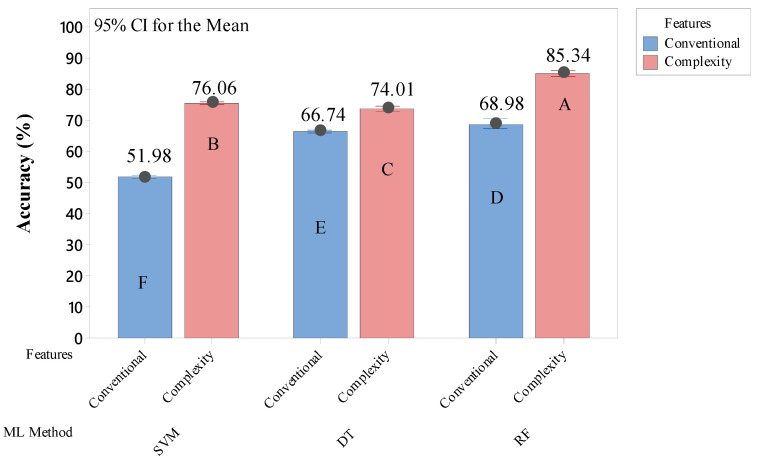
Accuracy comparison of AI models that are built using conventional and eye-movement complexity features. Means that do not share a letter are significantly different.

Based on the results above, the comparison between the model’s performances from conventional and eye-movement complexity features is shown in Figure 5 below. This figure shows that the models built using eye-movement complexity features resulted in significantly higher performance in detecting the computer activities. No matter what AI method was used to build the models, in both screened (*p*-value < 0.01) and all features (*p*-value < 0.05) groups, the accuracy from the eye-movement complexity features group consistently showed significantly higher. However, similar to the results above, the RF models resulted in the highest performance among the other models, significantly (*p*-value < 0.001).

## 4. Discussion

### 4.1. Roles of Screened Important Features to Help AI for Distinguishing the Computer Activities

The results of the accuracy detection indicate that screened important features have the ability to help AI models distinguish the different computer activities. The statistical analysis (ANOVA) selected the features that are more influenced by the different computer activities. For example, the ANOVA results show that left pupil diameters (LPD) were significantly influenced by the different computer activities, with watching activity causing the widest pupil diameter (19.93 pixels compared to 19.34 pixels in typing and 17.97 pixels in reading). The inclusion of conventional LPD to the important conventional eye-movement features helped the SVM model to improve its ability to discriminate the reading and watching activities. Table 4 describes how well the important conventional eye-movement features raise the accuracy in detecting the reading activity instead of confusing to watching activity, as happened in the RF model built using unscreened features (1.9% to 11.32%). This finding confirmed our previous study [8] and a study conducted by Yamada and Kobayashi [13], which also involved pupil dilation on the critical eye-movement features.

The decreasing trend of confusedness in other cells also indicated an improvement in detection performances. For example, the 1.47% data from reading activity were falsely detected as typing activity (RF all features group). This confusedness was decreased to 0.27% by using important features only. Moreover, the improvement was more obvious in complexity groups. Figure 5 shows that AI models, built using DT and RF methods, were improved after applying the important features as the predictors. The sensitivity of these AI models significantly increased after excluding the features that were not affected by different kinds of computer activities. These results confirm the recommendation in [8,41] to use ANOVA which proved to be the most potent selector on features engineering, specific for eye-movement data. However, the ANOVA selection method did not give significant help for SVM detection models, both in conventional and complexity groups. It confirmed certain findings that recommended SVM-based features selection methods, e.g., L-J (Lothar-Joachim) method [42,43], embedded features selection method [44], or Fisher method [45], to deal with biometric-based features used in SVM models.

### 4.2. Complexity Eye-Movement Features Potency for AI Modelling

The results that are shown in Figure 5 obviously describe how complexity features have strengthened the AI models to discriminate the different computer activities. Even though both use all features or only important features, eye-movement complexity features are consistently superior in detecting computer activities. The basic nature of complexity-based features that are containing “implicit” information about body responses to the human activity and states [46,47] has benefited AI models to help them to overcome the prediction confusedness (compare Table 4 with Table 5). As was expected, the findings in this study prove that complexity analysis is also suitable for eye-movement-based data, as useful as its usage in human heart rate [16], cerebral hemodynamics [17], blood pressure [18], and body movements [19,48] data. Moreover, the experimental procedure that resulted in moderate head movement also confirmed that AI models would be suitable for everyday use in distinguishing computer activities in daily life.

### 4.3. Contribution, Possible Applications in Healthcare, and Future Research

The results of this study were focused on showing how powerful eye-movement complexity features are for building AI models on human activity detection. The findings also stated that complexity analysis of eye-movement data is beneficial to describe the human body response. Specifically, these discoveries have contributed to promoting complexity analysis usage for showing the human body data changes that represent their real-time status during daily activities. The generated eye-movement complexity features are precious treasures for developing certain assistive healthcare systems and technologies that require distinguishable biological data in the near future. Moreover, the findings can be a strong foundation for further applications, e.g., eye fatigue, cognitive or mental workload, and emotional state detection for computer users. These states are affecting factors for human health conditions [7,49,50].

However, the relation between each eye-movement complexity feature to the different computer activities is not discussed yet in this study. The information related to this issue needs to be dug. It is recommended to find the statistical correlation between them in future works in order to find more precise predictors for specific computer activity, as was done on eye-movement conventional features [8,13]. Once it is done, the AI modeling experts in the healthcare field will have more understanding of human complexity changes in responding to the different computer activities. Regarding the experiment procedure, we suggest that the placement of two computer screens should be rearranged to minimize participant distraction in future works.

## 5. Conclusions

This follow-up study from previous work [8] shows the eye-movement complexity features potency to build AI models for detecting computer activities. By involving enough participants, the AI models that were built using eye-movement complexity features were able to detect three kinds of computer activities (reading, watching a video, and typing) with significantly better results than in the conventional eye-movement features group. The usage of ANOVA to select the important eye-movement features was also helpful in strengthening the AI models’ detection ability. However, the study had not explained the statistical relation between the humans’ complexity represented by their visions of the computer activities yet. Future works need to be performed to explore the correlation between them as the foundation for choosing more features correlated with the specific computer activities (e.g., reading, typing, watching, drawing, etc.) or any other occasion (e.g., emotion, fatigue, cognitive workload states).

## Figures and Tables

**Table 1:** The Architectures of AI Models.

No.	ML Method	Special Parameters
1	SVM	Default
2	DT	Max depth: 5; Min. samples leaf: 7
3	RF	Max depth: 5; n_estimators: 1000

**Table 2:** Screened conventional eye-movement features using the ANOVA method from the eye-movement features.

Features	Statistic						
	Mean	STD	Var	Median	Max	Min	Skew
FPOGD	***	***		***	***	***	**
LPD	***	***	***	***	***	***	
LPMM	***	***	***	***	***	***	*
RPD	***	***	***	***	***	***	
RPMM	***	***	***	***	***	***	***
BKDUR	***	***	***	***	***	***	***
BKPMIN							
SAC_MAG	***	***		***	***	***	***

* indicates *p*-value < 0.05; ** indicates *p*-value < 0.01; *** indicates *p*-value < 0.001; grey-color shaded cell indicates *p*-value > 0.05.

**Table 3:** Screened Eye-movement Complexity Features from CI of all IMF.

#of IMF Using CI	Eye-Movement Complexity Features								
	FPOGD	FPOGX	FPOGY	LPCX	LPCY	LPD	RPD	LPMM	RPMM
IMF 1	***	***	***		***	***	***	***	***
IMF 2	*	***	***	***	***	***	***	***	***
IMF 3	***	***	***	***	***	***	***	***	***
IMF 4	***	***	***	***	***	**	***		***
IMF 5	***	**		***		**	***	***	***
IMF 6	***				***		*	**	

* indicates *p*-value < 0.05; ** indicates *p*-value < 0.01; *** indicates *p*-value < 0.001; grey-color shaded cell indicates *p*-value > 0.05.

**Table 4:** Confusion matrices of the RF Models built both using all and important conventional eye-movement features.

RF All Conventional Eye-Movement Features (56)							RF Important Conventional Eye-Movement Features (45)		
Mean (*SD*)		Predicted			Mean (*SD*)		Predicted		
		Reading	Typing	Watching			Reading	Typing	Watching
Actual	Reading	1.90 (0.00)%	1.37 (0.00)%	26.89 (0.01)%	Actual	Reading	11.32 (0.03)%	0.27 (0.00)%	18.57 (0.04)%
	Typing	0.53 (0.00)%	29.31 (0.01)%	0.32 (0.00)%		Typing	0.44 (0.01)%	28.63 (0.07)%	1.08 (0.00)%
	Watching	0.32 (0.00)%	0.78 (0.01)%	29.05 (0.01)%		Watching	4.97 (0.01)%	0.60 (0.00)%	24.59 (0.01)%

The accuracy degree is denoted by shading, with lighter hues indicative of lower accuracy and darker hues indicating higher accuracy (white indicates 0% and red represents 100%).

**Table 5:** Confusion matrices of the DT and RF Models were built both using all-important complexity features.

**DT All Conventional** **Eye-movement Features (550)**					**DT Important Complexity** **Eye-movement Features (379)**				
**Mean (SD)**		**Predicted**			**Mean (SD)**		**Predicted**		
		**Reading**	**Watching**	**Reading**			**Reading**	**Watching**	**Typing**
Actual	Reading	18.81 (0.05)%	8.90 (0.02)%	2.46 (0.01)%	Actual	Reading	19.97 (0.05)%	8.57 (0.02)%	1.61 (0.00)%
	Watching	7.15 (0.02)%	20.80 (0.05)%	2.20 (0.01)%		Watching	7.76 (0.02)%	20.96 (0.05)%	1.44 (0.01)%
	Typing	1.62 (0.00)%	2.08 (0.01)%	26.46 (0.01)%		Typing	1.97 (0.01)%	1.42 (0.01)%	26.77 (0.07)%
**RF All Conventional** **Eye-movement Features (550)**					**RF Important Complexity** **Eye-movement Features (379)**				
**Mean (SD)**		**Predicted**			**Mean (SD)**		**Predicted**		
		**Reading**	**Watching**	**Typing**			**Reading**	**Watching**	**Typing**
Actual	Reading	22.18 (0.06)%	6.85 (0.01)%	1.13 (0.00)%	Actual	Reading	23.89 (0.06)%	5.48 (0.48)%	0.73 (0.01)%
	Typing	5.03 (0.01)%	24.42 (0.07)%	0.71 (0.00)%		Typing	4.58 (0.01)%	24.69 (0.07)%	0.91 (0.00)%
	Watching	0.15 (0.00)%	0.34 (0.00)%	29.67 (0.07)%		Watching	0.23 (0.02)%	0.20 (0.00)%	29.75 (0.08)%

The accuracy degree is denoted by shading, with lighter hues indicative of lower accuracy and darker hues indicating higher accuracy (white indicates 0% and red represents 100%).
